# Interactive effect of dietary calcium and phytase on broilers challenged with subclinical necrotic enteritis: part 2. Gut permeability, phytate ester concentrations, jejunal gene expression, and intestinal morphology

**DOI:** 10.1016/j.psj.2020.06.030

**Published:** 2020-07-03

**Authors:** H.K. Zanu, S.K. Kheravii, N.K. Morgan, M.R. Bedford, R.A. Swick

**Affiliations:** ∗ School of Environmental and Rural Science, University of New England, Armidale, NSW 2351, Australia; † AB Vista, Marlborough, Wiltshire SN8 4AN, United Kingdom

**Keywords:** dietary calcium, gut health, necrotic enteritis, phytate esters and phytase

## Abstract

Calcium has the capacity to interact with phytate-P to form Ca-phytate complexes and decrease the ability of exogenous phytase to degrade phytic acid. This study investigated the hypothesis that high dietary Ca would impair gut permeability, phytate esters (inositol x-phosphate, IPx: IP3, IP4, IP5, and IP6) degradation, jejunal gene expression, and intestinal morphology. Ross 308 day-old male broilers (n = 768) were distributed into 48-floor pens each housing 16 birds in a factorial arrangement. Factors were NE challenge—no or yes; phytase level of 500 or 1,500 FTU/kg, and Ca level 0.6 or 1.0% starter, 0.5 or 0.9% grower, 0.4 or 0.8% finisher with available P in each phase. Challenged birds were gavaged with 3 field strains of Eimeria on day 9 and 10^8^ CFU per mL of *Clostridium perfringens* Strain EHE-NE18 on day 14 and day 15. A phytase × Ca interaction was observed in the ileum for IP3 (*P* < 0.01), IP4 (*P* < 0.05), and IP6 (*P* < 0.01). The IP3 and IP4 concentrations were similar for both doses of phytase in the presence of low Ca, but with high Ca, both increased significantly but to a greater extent when the high dose of phytase was used. While IP6 concentrations were low and similar between both doses of phytase at low Ca levels, increasing dietary Ca levels increased IP6 concentrations regardless of phytase dose, but the effect was greater in the low phytase diet. A phytase × Ca interaction was detected for vitamin D receptor (VDR) (*P* < 0.05) expression where bird fed low phytase and low Ca recorded the highest expression of VDR, all other treatments being equivalent. The challenge decreased crypt depth to villus height ratio (*P* < 0.001). Challenge birds had higher fluorescein isothiocyanate dextran (*P* < 0.05) in blood compared with unchallenged birds. Thus, high Ca and high phytase, while not the best for IP6 destruction, did not lead to huge reductions in indicators of gut health.

## Introduction

There has been a spate of research about the effect of exogenous phytase in chicken nutrition in recent times, resulting in the heightened application in the poultry industry because of its proven beneficial impacts on the utilization of feed ingredients containing phytic acid (Angel et al., 2002; Wu et al., 2004; dos Santos et al., 2014; Li et al., 2017; Walk and Olukosi, 2019). Phytic acid is naturally present in most chicken diets at concentrations of approximately 1%. The negative effects of phytic acid on nutrient availability has been widely studied (Adeola and Sands, 2003; Selle et al., 2006; Cowieson et al., 2008), including its impact on reducing pepsin and pepsinogen activity (Woyengo et al., 2010; Yu et al., 2012). The inhibition of the conversion of pepsinogen to pepsin by phytic acid results in the greater secretion of acid to maintain gastric digestion. The gastric region, therefore, has to produce more mucin to protect itself as does the duodenum to prevent damage on receipt of the digesta which is more acidic than usual, thus increasing endogenous losses of nutrients (Rutherfurd et al., 2004; Walk and Olukosi, 2019). The current focus of the research is on factors that limit the efficacy of phytase (Sebastian et al., 1996; Driver et al., 2005; Pillai et al., 2006; Kim et al., 2018). Many research findings also demonstrate the benefits of phytase in improving nutrient utilization (Martinez-Amezcua et al., 2006; Amerah et al., 2014), gut microbiome (Borda-Molina et al., 2019), immune function (Liu et al., 2008), bone mineralization (Adeola and Walk, 2013), and the ultimate productive performance of broilers fed diet expected to contain phytic acid. But, the efficacy of phytases can be limited by various factors including nutritional imbalances. One such limiting factor is dietary Ca (Bedford and Rousseau, 2017).

Phytate is a polyanionic molecule with the capacity to chelate multivalent cations, including Ca, to form mineral phytate complexes that resists the hydrolytic capacity of phytase. The complex formation is facilitated by high concentrations of Ca and phytate and elevated gut pH (Selle et al., 2009). The pH in the digesta ranges from highly acid in the gizzard and proventriculus to approaching neutrality in the intestines and ceca. Thus, there is a reduction in the binding between positively charged Ca ions and negatively charged phytate molecules in the gastric phase and increased chelation between these constituents at a higher pH in the intestines (Selle et al., 2009).

Calcium also can interact with P in the gut to form either Ca-phytate complex or Ca phosphate thereby reducing their disappearance (Tamim et al., 2004; Sommerfeld et al., 2018). Calcium in the form of limestone or monocalcium phosphate/monodicalcium phosphate is a potent buffer, and therefore, a high concentration of Ca has a negative effect on the release of P through increased gizzard pH, precipitation of P, and interaction with phytate, rendering it less accessible. It is suggested that the addition of phytase to diets with a high Ca to P ratio may recover some of the loss in bird performance (Bedford and Rousseau, 2017). The complete dephosphorylation of inositol hexaphosphate (**IP6**) by phytase to the lower esters: inositol pentaphosphate (**IP5**), inositol tetraphosphate (**IP4**), inositol triphosphate (**IP3**), inositol biphosphate (**IP2**), and inositol monophosphate (**IP1**) has been demonstrated to promote growth performance (Zyła et al., 2004). However, a wider Ca:P ratio reduce the disappearance of the lower phytate esters in the digestive tract of broilers (Applegate et al., 2003).

High levels of dietary Ca level may impact necrotic enteritis (**NE**) by increasing the growth of *Clostridium perfringens* as this bacterium is acid sensitive and opportunistic, multiplying faster under conditions of high gut pH and an abundance of undigested nutrients, particularly amino acids, which high dietary Ca promotes. Thus, Ca is a contributor to the pathogenesis of NE (Williams, 2005). Also, NetB toxin produced by *C. perfringens* type A is believed to be dependent on Ca (Keyburn et al., 2010). Necrotic enteritis has been reported to have a negative effect on the morphology of the gut (Jayaraman et al., 2013). A high villus: crypt ratio indicates a matured and functional epithelium. A shallow crypt depth allows for constant cell renewal, and deeper crypts indicate fast tissue turnover in response to increased requirements for maintenance of the digestive tract and gut barrier integrity (Guo and Guo, 2012). In addition, many nutrient transporters and receptors are expressed in the brush-border membranes of the small intestinal epithelium. Thus, a disruption of the brush border might affect the expression of these genes. Therefore, the objective of this study was to determine the effect of 2 Ca to P ratios and 500 vs. 1,500 FTU/kg of phytase on phytate esters degradation, gut permeability, jejunal gene expression, and intestinal morphology of broiler chickens challenged with subclinical NE.

## Materials and methods

### Birds and Management

All experimental procedures were reviewed and approved by the University of New England Animal Ethics Committee. A total of 768 of day-old Ross 308 male chicks were weighed and randomly allocated to 48-floor pens (0.85 m^2^) with 6 replicate pens per treatment and 16 birds per pen. New softwood shavings were used as bedding material of about 8 cm deep in each pen. Each pen was fitted with a single tube feeder (32 cm diameter) and 4 nipple drinkers. The lighting and temperature program during the experimental period followed the Ross 308 guidelines (Aviagen, 2014)

### Diet Composition

Four diets were formulated largely following Ross 308 nutrient specifications for digestible amino acids and MEn but slightly modified to align with local industry practice. Treatments were arranged in a 2 × 2 × 2 factorial arrangement. Factors were calcium, 0.6 or 1.0%, starter (**S**); 0.5 or 0.9%, grower (**G**); and 0.4 or 0.8%, finisher (**F**); phytase, 500 or 1,500 FTU/kg (Quantum Blue, AB Vista, Malborough, UK); and NE challenge (no or yes). The higher dose was selected as it is commonly used in industry. The recommended matrix values for the 500 FTU dose were used for both levels of phytase when formulating the diets that is Ca, P, Na, ME, CP, arginine, lysine, methionine, methionine + cystine, tryptophan, isoleucine, threonine, and valine values of 1,650, 1,500, 350%, 520,000 kcal/kg, 4,210, 130, 170, 39, 390, 190, 255, 330, and 230%, respectively (amino acids expressed as standardized ileal digestible were applied to the phytase dose based on manufacturers recommendations. The phytase used was an *Escherichia coli* 6-phytase expressed in *Trichoderma reesei*. The available P was formulated to be at the same level irrespective of the Ca level at 0.40% S, 0.35% G, and 0.35% F. The diet with high dietary Ca had a Ca: P ratio of 2.5, whereas those with lower dietary Ca had a narrower ratio of 1.5. The diets were offered *ad libitum* throughout the S (day 0 to 14), G (day 14 to 28), and F (day 28 to 42) phases. The S diets were offered in a crumbled form while the G and F diets were mixed and pelleted at 65°C. The diet compositions are shown in Table 1. The recovered phytate esters (analyzed) of the experimental diets are presented in Table 2.

**Table 1:** Ingredient and nutrient composition of basal diets (g/kg), as-fed.

Ingredients	Starter (0-14 D)				Grower (14-28 D)				Finisher (28-42 D)			
	0.6% Ca + low phytase	0.6% Ca + high phytase	1.0% Ca + low phytase	1.0% Ca + high phytase	0.6% Ca + low phytase	0.6% Ca + high phytase	1.0% Ca + low phytase	1.0% Ca + high phytase	0.6% Ca + low phytase	0.6% Ca + high phytase	1.0% Ca + low phytase	1.0% Ca + high phytase
Wheat	620	620	597	597	708	708	687	687	723	723	702	702
SBM	296	296	302	302	185	185	189	189	166	166	170	170
Canola expeller cold press	50	50	50	50	70	70	70	70	70	70	70	70
Canola oil	12	12	18	18	14	14	21	21	23	23	30	30
Limestone	6	6	16	16	5	5	15	15	3	3	14	14
MDC phosphate^1^	4.19	4.19	4.26	4.26	2.42	2.42	2.5	2.5	0.64	0.64	0.71	0.71
Xylanase^2^	0.1	0.1	0.1	0.1	0.1	0.1	0.1	0.1	0.1	0.1	0.1	0.1
Phytase^3^	0.1	0.3	0.1	0.3	0.1	0.3	0.1	0.3	0.1	0.3	0.1	0.3
Salt	1.41	1.41	1.43	1.43	0.83	0.83	0.85	0.85	0.82	0.82	0.84	0.84
Na bicarbonate	2	2	2	2	2	2	2	2	2	2	2	2
TiO_2_	-	-	-	-	5	5	5	5	5	5	5	5
Vitamins^4^	1	11	1	1	0.7	0.7	0.7	0.7	0.5	0.5	0.5	0.5
Trace minerals^5^	1.2	1.2	1.2	1.2	1	1	1	1	1	1	1	1
Choline Cl 60	0.64	0.64	0.67	0.67	0.74	0.74	0.77	0.77	0.58	0.58	0.6	0.6
L-lysine HCl	2.34	2.34	2.24	2.24	2.81	2.81	2.75	2.75	2.61	2.61	2.55	2.55
DL-methionine	1.99	1.99	2	2	1.58	1.58	1.6	1.6	4.89	4.89	4.9	4.9
L-threonine	0.54	0.54	0.53	0.53	0.57	0.57	0.58	0.58	0.51	0.51	0.51	0.51
Nutrients												
MEn, kcal/kg	3,000	3,000	3,000	3,000	3,100	3,100	3,100	3,100	3,100	3,100	3,100	3,100
Crude protein, %	24.4	24.4	24.3	24.3	20.9	20.9	20.8	20.8	20.3	20.3	20.3	20.3
SID, %												
Arginine	1.40	1.40	1.41	1.41	1.14	1.14	1.14	1.14	1.09	1.09	1.09	1.09
Lysine	1.24	1.24	1.24	1.24	1.05	1.05	1.05	1.05	0.99	0.99	0.99	0.99
Methionine	0.5	0.5	0.5	0.5	0.43	0.43	0.43	0.43	0.75	0.75	0.75	0.75
d M + C	0.89	0.89	0.88	0.88	0.80	0.80	0.80	0.80	1.11	1.11	1.11	1.11
Tryptophan	0.27	0.27	0.27	0.27	0.23	0.23	0.23	0.23	0.22	0.22	0.22	0.22
Isoleucine	0.88	0.88	0.88	0.88	0.73	0.73	0.73	0.73	0.70	0.70	0.70	0.70
Threonine	0.79	0.79	0.79	0.79	0.68	0.68	0.68	0.68	0.65	0.65	0.65	0.65
Valine	0.98	0.98	0.98	0.98	0.84	0.84	0.84	0.84	0.81	0.81	0.81	0.81
Calcium, %	0.60	0.60	1.00	1.00	0.51	0.51	0.91	0.91	0.43	0.43	0.83	0.83
Available P, %	0.40	0.40	0.40	0.40	0.36	0.36	0.36	0.36	0.32	0.32	0.32	0.32
Sodium, %	0.18	0.18	0.18	0.18	0.16	0.16	0.16	0.16	0.16	0.16	0.16	0.16
Choline, pmm	1,700	1,700	1,700	1,700	859	859	885	885	731	731	757	757
Analyzed, DM basis												
Calcium, %	0.51	0.52	0.77	0.84	0.40	0.40	0.82	0.92	0.38	0.33	0.76	0.79
Total P, %	0.59	0.60	0.59	0.61	0.54	0.54	0.58	0.59	0.51	0.52	0.52	0.51

Abbreviation: SBM, soybean meal; SID, standardised ileal digestibility.

1 MDC phosphate or monodicalcium phosphate contains 21% P and 16% Ca, Kynophos 21, sourced from BEC, Brisbane, QLD.

2 Xylanse was Econase XP 25, providing 160,000 birch xylanase units per gram inclusion, AB Vista Feed Ingredients, UK, no matrix values applied.

3 Phytase was Quantum Blue 5G, AB Vista Feed Ingredients, UK to provide 500 FTU/kg (0.1% phytase) or 1,500 FTU/kg (0.3% phytase). Matrix values used Ca, P, Na, ME, CP, arginine, lysine, methionine, methionine + cystine, tryptophan, isoleucine, threonine, and valine of 1,650, 1,500, 350%, 520,000 kcal/kg, 4,210, 130, 170, 39, 390, 190, 255, 330, and 230%, respectively (amino acids expressed as SID).

4 Vitamin premix per kg diet: vitamin A, 12 MIU; vitamin D, 5 MIU; vitamin E, 75 mg; vitamin K, 3 mg; nicotinic acid, 55 mg; pantothenic acid, 13 mg; folic acid, 2 mg; riboflavin, 8 mg; cyanocobalamin, 0.016 mg; biotin, 0.25 mg; pyridoxine, 5 mg; thiamine, 3 mg; antioxidant, 50 mg.

5 Trace mineral concentrate supplied per kilogram of diet: Cu (sulfate), 16 mg; Fe (sulfate), 40 mg; I (iodide), 1.25 mg; Se (selenate), 0.3 mg; Mn (sulfate and oxide), 120 mg; Zn (sulfate and oxide), 100 mg; cereal-based carrier, 128 mg; mineral oil, 3.75 mg.

**Table 2:** Recovered phytate esters and inositol concentration (μmol/g DM) in the in experimental diets.

Treatment				Starter					Grower					Finisher				
				IP3	IP4	IP5	IP6	Inositol	IP3	IP4	IP5	IP6	Inositol	IP3	IP4	IP5	IP6	Inositol
	NE	Phy	Ca															
1.	-	500	Low	0.154	0.588	2.466	16.757	0.572	0.104	0.714	3.610	14.857	0.092	0.252	1.343	6.582	12.044	0.052
2.	-	1,500	Low	0.064	0.597	2.743	17.181	0.000	0.181	0.672	3.873	15.030	0.009	0.280	1.461	6.690	11.591	0.051
3.	-	500	High	0.133	0.651	2.801	17.032	0.341	0.132	0.845	4.209	16.516	0.245	0.170	1.417	7.018	11.576	0.094
4.	-	1,500	High	0.131	0.762	2.984	16.500	0.299	0.147	0.805	4.078	14.654	0.195	0.244	1.532	6.977	11.612	0.049
5.	+	500	Low	0.156	0.770	3.138	16.685	0.350	0.143	0.794	4.274	15.163	0.070	0.192	1.671	7.578	11.309	0.052
6.	+	1,500	Low	0.281	0.736	3.033	15.455	0.283	0.060	1.026	5.543	13.607	0.196	0.196	1.630	7.249	10.922	0.051
7.	+	500	High	0.205	0.846	3.319	15.460	0.333	0.104	1.148	5.540	12.907	0.112	0.176	1.737	7.585	11.141	0.094
8.	+	1,500	High	0.155	0.832	3.356	15.884	0.106	0.116	1.173	5.829	12.612	0.222	0.167	1.855	7.874	10.495	0.049

Phytase (Quantum Blue 5G).

Abbreviations: Ca, calcium; IP3, inositol triphosphate; IP4, inositol tetraphosphate; IP5, inositol pentaphosphate; IP6, inositol hexaphosphate; NE, necrotic enteritis; phy, phytase.

### Challenge

The NE challenge was performed in accordance with reported procedures (Stanley et al., 2014; Rodgers, et al., 2015). Half of the birds (384) were challenged *per os* with 5,000 oocysts of field strains of *Eimeria acervulina* and *Eimeria maxima* and 2,500 oocytes of *Eimeria brunetti* (Eimeria Pty Ltd) on day 9 to increase the outcome of the NE and 10^8^ CFU per mL of *C. perfringens* Strain EHE-NE18 (known to express NetB toxin, CSIRO) on day 14 and day 15.

### Fluorescein Isothiocyanate Dextran

Two birds (of average weight) per pen were gavaged with fluorescein isothiocyanate dextran solution (**FITC-d**; Sigma-Aldrich, Stockholm, Sweden; average mol weight of 4,000 and FITC-d: glucose of 1:250) (4.17 mg/kg bird dissolved in water) on day 16, 180 min before collection of blood, to evaluate the permeability of the intestinal barrier following the procedure of Barekatain et al. (2019) with little modification. Blood samples were taken by stunning the birds with an electric stunner with blood collected from the jugular vein after decapitation. The blood samples were centrifuged at 3,000 × g for 15 min, and serum samples collected. The serum was diluted in phosphate buffer saline (1:1). Fluorescence was measured at 485 nm excitation and 528 nm emission using SpectraMax M2e Microplate Reader (Molecular Devices, San Jose CA). Levels of fluorescence in the samples were converted to respective FITC-d microgram per milliliter of serum based on a calculated standard curve.

### Phytate Esters Determination

The determination of phytate and its intermediate derivatives was done using the procedures of Walk et al. (2018). Briefly, on day 29 posthatch, freeze-dried gizzard and ileal digesta samples collected from the 2 birds used for the FITC-d determination were extracted with 10 mL of 0.5 mol HCl for 1 h at 20°C by ultrasonication. The extracts were then centrifuged for 10 min at 2,200 × g, and 5 mL of the supernatant was evaporated to dryness in a vacuum centrifuge. The samples were then re-dissolved in 1 mL of distilled, deionized water by ultrasonication for 1 h at 20°C and centrifuged for 15 min at 18,000 × g. The resulting supernatant was filtered through a 13-mm syringe filter with a 0.45 μm membrane (GH Polypro Acrodisc; Pall Corporation, Ann Arbor, MI) and placed in a 30 kDa centrifugal filter (Microcon Ultracel YM30; Millipore Corporation, Bedford, MA) and finally centrifuged for 30 min at 9,000 × g. Quantification of inositol phosphates (IP3 to IP6) was performed using high-performance ion chromatography and UV detection at 290 nm after postcolumn derivatization. Myoinositol was determined using high-performance liquid chromatography with pulsed amperometric detection.

### RNA Extraction and cDNA Synthesis

For RNA extraction, the jejunal tissues from the birds sampled for the phytate esters determination were rinsed with chilled sterile phosphate-buffered saline and immediately placed in RNAlater, stored at 4°C for 4 to 8 h before storage at −20°C until further use. Approximately, 80 mg of the sample tissue was homogenized in 1 mL TRIsure (Bioline, Sydney, Australia) using an IKA T10 basic Homogenizer (Wilmington, NC), and total RNA was extracted as per manufacturer's instructions. Total RNA was purified using ISOLATE II RNA Mini Kit (Bioline) as per the manufacturer's instructions. RNA purity and concentration were measured using the NanoDrop ND-8000 spectrophotometer (Thermo Fisher Scientific, Waltham, MA). The integrity of the RNA samples was evaluated in Agilent 2100 Bioanalyzer (Agilent Technologies, Inc., Waldbronn, Germany) using RNA 6000 Nano kit (Agilent Technologies, Inc., Palo Alto, CA) as per the manufacturer's instructions. The samples with RNA integrity number (RIN) > 7.5 were used in this study as they were considered as sufficient. The extracted RNA of each sample was reverse-transcribed to cDNA with the SensiFAST cDNA Synthesis Kit (Bioline) as per the manufacturer's instructions. The Rotorgene 6000 real-time PCR machine (Corbett, Sydney, Australia) was used to convert the RNA into cDNA. Synthesized cDNA samples were diluted 1:10 with nuclease-free water and stored at −20 C until used.

### Primer Sources

The primers used in this study were either sourced from previously published papers (Table 3) or were designed with NCBI primer tool (https://www.ncbi.nlm.nih.gov). Agilent 2100 Bioanalyzer (Agilent Technologies Inc.) was used to determine the primer specificity for each pair with Agilent DNA 1000 Kit (Agilent Technologies, Inc.). Only the specific primer pairs with high efficiency (in terms of high RNA integrity) were used in this study.

**Table 3:** Sequences of primers used for quantitative real-time PCR.

Gene symbol	Group	Gene name	Primer sequence (5ʹ-3ʹ)	Ta°C	Amplicon size (bp)	Reference	Function
CLDN1	Tight junction protein	Claudin 1	F-CTTCATCATTGCAGGTCTGTCAG R-AAATCTGGTGTTAACGGGTGTG	60	103	This study^1^	Maintenance of intestinal barrier function
CLDN5	Tight junction protein	Claudin 5	F-GCAGGTCGCCAGAGATACAG R-CCACGAAGCCTCTCATAGCC	61	162	This study	Maintenance of intestinal barrier function
JAM2	Tight junction protein	Junctional adhesion molecule 2	F-AGACAGGAACAGGCAGTGCTAG R-ATCCAATCCCATTTGAGGCTAC	60	135	This study	Maintenance of intestinal barrier function
OCLD	Tight junction protein	Occludin	F- ACGGCAGCACCTACCTCAA R- GGGCGAAGAAGCAGATGAG	60	123	(Du et al., 2016)	Maintenance of intestinal barrier function
NaPi-IIb	Phosphorus transporter	Na-dependent Pi cotransporters, type IIb	F- CGGTCCGTTCACTCTGTTGC R- GAAGCCACGTTGCCTTTGTG	60	165	This study	Mediation of intestinal phosphate uptake
VDR	Vitamin D transporter	Vitamin D receptor	F- ACGCAGACATGGACACCGT R- GGACAGGTGAACATCGCTTTC	60	193	This study	Provides instructions for making vitamin D receptor
CALB1	Calcium transporter	Calbindin 1	F- GGCAGGCTTGGACTTAACACC R- GTCGGCAACACCTGAGCAAG	60	105	This study	Transport protein of calcium
ATP1A1	Calcium transporter	ATPase Na+/K+ transporting subunit alpha 1	F- GTCAACCCGAGGGATGCTAA R- ACTGCTACAATGGCACCCTG	60	179	(Kheravii et al. 2018)	Provides instructions for making Na^+^/K^+^ ATPase
ATP5A1W	Calcium transporter	ATP synthase subunit alpha	F- GGCAATGAAACAGGTGGCAG R- GGGCTCCAGCTTGTCTAAGTGA	60	232	This study	Synthesis of ATP
ATP13A4	Calcium transporter	ATPase type 13A4	F-CCAAAGCTCCTGCTAAATGC R-ATGCCTCCTGCTCTGACAGT	61	178	(Lee and Kim, 2018)	
ATP2A1	Calcium transporter	ATPase, Ca++ transporting, cardiac muscle, fast twitch 1	F-CGCTGTCAATCAGGACAAGA R-GTCGTTAAAGTGGCCGATGT	61	250	(Lee and Kim, 2018)	Catalyzes the hydrolysis of ATP and translocation of calcium from the cytosol to the sarcoplasmic reticulum lumen.
ATP2B1	Calcium transporter	ATPase, Ca++ transporting, plasma membrane 1	F-CTGGGCATGGGAACACTACT R-CACGACGTAATTCTCGCTCA	60	169	(Lee and Kim, 2018)	Catalyzes the hydrolysis of ATP and maintenance of intracellular calcium homeostasis
CASR	Calcium transporter	Calcium sensing receptor	F-CTGCGTGATTTGGCTCTACA R-GGCAAAGAAGAAGCAGATGG	60	160	(Lee and Kim, 2018)	Regulator of parathyroid hormone synthesis and secretion and systemic calcium homeostasis.
SLC8B1	Calcium transporter	(Sodium/lithium/calcium exchanger), member B1	F-CTTCGAGCTGAGCAACACTG R-CCCACACCCACCAGAATATC	60	169	(Lee and Kim, 2018)	Mediates mitochondrial calcium extrusion and mitochondrial calcium homeostasis
CACNA1A	Calcium channel	Calcium channel, voltage-dependent, P/Q type alpha 1A subunit	F-GCAGCGGGTCTATAAGCAGT R-GCGATGATGGTGGCTAAAAT	60	198	(Lee and Kim, 2018)	Provides instructions for making calcium channels
TPCN2	Calcium channel	Two pore segment channel 2	F-AGGTGCTGTGGTTCCTATGG R-GCCCACGGACTTGTGTATCT	60	210	(Lee and Kim, 2018)	Very little is known about the physiological functions but believe to form functional Ca^2+^-permeable channel
TPCN3	Calcium channel	Two-pore calcium channel 3	F-TGGGAATGGGAGTTCAAGAG R-CTGCCTCAAACATACGCTGA	60	183	(Lee and Kim, 2018)	Very little is known about the physiological functions but believe to form functional Ca^2+^-permeable channel
MUC-2	Inflammatory genes	Mucin 2	F- CCCTGGAAGTAGAGGTGACTG R- TGACAAGCCATTGAAGGACA	60	143	(Fan et al., 2015)	The physical and biological barrier protecting mucous epithelia
RPL4	Housekeeping genes	Ribosomal protein L4	F: TTATGCCATCTGTTCTGCC R: GCGATTCCTCATCTTACCCT	60	235	(Yang et al., 2013)	Component of the 60S subunit and encodes a ribosomal protein
β-actin	Housekeeping genes	β-actin	F-CTCTGACTGACCGCGTTACTCC R-CCATACCAACCATCACACCCTG	60	175	This study	Cytoskeletal structural protein, nucleotide, and ATP binding

1 Primer sequencing for the gene was done in the present study and not that reported in a published paper.

### Quantitative Real-Time Polymerase Chain Reaction

Quantitative real-time PCR was performed using Rotorgene 6000 real-time PCR machine (Corbett Research, Sydney, Australia). The geNorm module in qbase + software was employed to determine 2 most stable genes among 7: Ribosomal protein L4, β-actin, glyceraldehyde 3-phosphate dehydrogenase, hypoxanthine-guanine phosphoribosyltransferase, hydroxymethylbilane synthase, thyroxine-binding protein; tyrosine 3-monooxygenase/tryptophan 5-monooxygenase activation protein zeta different reference genes. Based on the expression stability ribosomal protein L4 and β-actin were used to normalize the expression of target genes in the jejunum (Table 3). The relative quantification of genes using arithmetic mean method in qbase+ was exported to SAS 9.3 package (SAS Institute Inc., 2010) for further analysis.

### Morphometrics of Jejunum Mucosa

Jejunal samples were taken from the 2 birds from which blood was collected and fixed in 10% buffered formalin until ready for processing following the methods of Xue et al. (2018). The tissues samples were processed in Leica TP 1020 Tissue Processor (GMI Inc., Ramsey, MN) according to the program as follows: 30% ethanol for 2 h; 50% ethanol for 2 h; 70% ethanol for 2 h; 80% ethanol for 2 h; 95% alcohol for 1 h; absolute ethanol for 1 h; absolute ethanol for 1 h; 50:50 ethanol: xylol for 1 h; xylol for 1 h; xylol for 1 h; paraplast + Vac for 2 h, and paraplast + Vac for 2 h. Tissue blocks were prepared for sectioning, and each block was cut into 6 μm cross-section thickness and mounted on glass slides. The slides were then stained using the Harris's hematoxylin and eosin staining method. The cross-sections were viewed by light microscopy (Olympus CX41 microscope) using a 10 × objective and color images captured with the software Analysis 5.0 (Olympus Soft Imaging Solutions GmbH, Münster, Germany). Five muscularis layer, villi height, total height (muscularis layer + villi height), crypt depth, basal width, and apical width per section, and 4 sections per sample were analyzed. The apparent villi surface area was calculated as: (basal width + apical width)/2 × villus length.

### Statistical Analyses

The data were evaluated as a fixed-effect model using the effects described in the statistical model, as follows:

$${\text{Y}}_{\text{ijkl}}\;=\;\mu \;+\;NE_{\text{i}}\;+\;{\text{P}}_{\text{j}}\;+\;Ca_{k}\;+\;{\left(NE*P\right)}_{ij}\;+\;{\left(NE*Ca\right)}_{ik}\;+\;{\left(P*Ca\right)}_{jk}\;+\;\left(NE*P*Ca\right)i_{jk}\;+\;e_{ijkl}$$

where, *Y*_*ij*_ is the response expected independent variables, μ = overall mean, NE_i_ = fixed effect of necrotic enteritis (i = challenged or not challenged), P_j_ = the fixed effect of phytase (j = low or high), Ca_k_ = the fixed effect of calcium (k = low or high), (NE∗P)_ij_ = interaction between NE and phytase, (NE∗Ca)_ik_ = interaction between necrotic enteritis and Ca, (P∗Ca)_jk_ = interaction between phytase and calcium, (NE∗P∗Ca)_ijk_ = is the 3-way interaction and *e*_*ij*_ is the random residual error ∼ *N* (0, s2e).

The study used a completely randomized design with data analyzed as a 2 × 2 × 2 factorial arrangement of treatments using the Minitab 19 statistical software to assess the main effects and 2 or 3-way interactions, with the factors as challenge (no or yes), phytase (500 or 1,500 FTU/kg), and Ca (low or high). Tukey's mean separation test was used to make pairwise comparisons between treatment means (*P* < 0.05). The Box-Cos transformation of the Minitab 19 statistical software was used to test and confirm the normality of all the data before analysis. The statistical unit for the data collected was the average of the sampled birds.

## Results

### Phytate Esters Concentration in the Gizzard and Ileum, Day 29

The challenge as main effect increased (*P* < 0.01) inositol in the gizzard (Table 4). The high level of phytase increased IP3 (*P* < 0.001) but decreased IP4 (*P* < 0.001) and IP5 (*P* < 0.05). Dietary Ca as a main effect decreased inositol (*P* < 0.05). There was no significant main, 2-way, or treatment effect on IP6 concentration in the gizzard.

**Table 4:** Effect of necrotic enteritis, phytase, and calcium on phytate esters and inositol concentration (μmol/g DM) in the gizzard digesta of broilers 29 D posthatch.

Effects				Inositol	IP3	IP4	IP5	IP6
	NE	Phy	Ca					
Main effects								
NE	-			2.404^**b**^	0.610	1.221	0.219	0.365
	+			3.840^**a**^	0.648	1.424	0.176	0.350
Phy		500		3.044	0.388^**b**^	2.038^**a**^	0.262^**a**^	0.441
		1,500		3.201	0.870^**a**^	0.607^**b**^	0.133^**b**^	0.275
Ca			Low	3.547^**a**^	0.603	1.389	0.177	0.441
			High	2.697^**b**^	0.655	1.256	0.218	0.274
SEM				1.32	0.16	0.06	0.11	0.15
P > f								
NE				0.001	0.614	0.477	0.510	0.872
Phy				0.696	0.001	0.001	0.049	0.090
Ca				0.040	0.492	0.641	0.523	0.088
NE × Phy				0.784	0.161	0.192	0.261	0.808
NE × Ca				0.464	0.775	0.422	0.595	0.644
Phy × Ca				0.843	0.626	0.766	0.568	0.654
NE × Phy × Ca				0.622	0.203	0.411	0.231	0.496

^a,b,c^Means in the same column with different superscripts are significantly different (*P* < 0.05).

Phytase (Quantum Blue 5G).

2 or 3-way interaction by Tukey.

Abbreviations: Ca, calcium; IP3, inositol triphosphate; IP4, inositol tetraphosphate; IP5, inositol pentaphosphate; IP6, inositol hexaphosphate; NE, necrotic enteritis; phy, phytase.

A challenge × Ca interaction (*P* < 0.05) was detected in the ileum for IP5, where the IP5 concentration was lower in birds fed the lower level of dietary Ca, in both challenged and unchallenged birds (Table 5). In birds fed the high Ca diet, IP5 was greater in unchallenged birds. A phytase × Ca interaction was observed in the ileum for IP3 (*P* < 0.01), IP4 (*P* < 0.05), and IP6 (*P* < 0.01). Inositol triphosphate and IP4 concentrations were similar for both doses of phytase in the presence of low Ca but with high Ca both increased to a greater extent when the high dose of phytase was used. While IP6 concentrations were low and similar between both doses of phytase at low Ca levels, increasing dietary Ca level markedly increased IP6 concentrations regardless of phytase dose, but the effect was far greater in the low phytase diet.

**Table 5:** Effect of necrotic enteritis, phytase, and calcium on phytate esters and inositol concentration (μmol/g DM) in the ileal digesta of broilers 29 D posthatch.

Effects				Inositol	IP3	IP4	IP5	IP6
	NE	Phy	Ca					
2-Way interactions								
NE∗Ca								
-			Low	23.90	0.368	1.790	0.554^c^	3.52
-			High	13.50	1.982	7.194	5.143^a^	17.46
+			Low	36.02	0.245	1.425	0.278^c^	2.41
+			High	24.64	1.131	4.759	2.656^b^	10.53
Phy∗Ca								
		500	Low	27.97	0.345^b^	1.885^b,c^	0.397	3.88^b,c^
		500	High	17.15	1.009^b^	4.681^a,b^	4.779	19.53^a^
		1,500	Low	31.95	0.269^b^	1.330^c^	0.435	2.04^c^
		1,500	High	20.99	2.103^a^	7.272^a^	3.021	8.45^b^
Main effects								
NE	-			18.70^b^	1.175^a^	4.492	2.849	10.49^a^
	+			30.33^a^	0.688^b^	3.092	1.467	6.47^b^
Ca			Low	29.96^a^	0.307	1.608	0.416	2.96
			High	19.07^b^	1.556	5.976	3.900	13.99
SEM				5.88	0.72	2.32	1.38	4.15
P > f								
NE				0.000	0.026	0.066	0.016	0.020
Phy				0.092	0.021	0.178	0.124	0.000
Ca				0.000	0.000	0.000	0.000	0.000
NE × Phy				0.094	0.273	0.875	0.571	0.456
NE × Ca				0.829	0.092	0.171	0.050	0.087
Phy × Ca				0.974	0.008	0.040	0.109	0.008
NE × Phy × Ca				1.000	0.279	0.587	0.343	0.322

^a,b,c^Means in the same column with different superscripts are significantly different (*P* < 0.05).

Phytase (Quantum Blue 5G).

2-way or 3-way interaction by Tukey.

Abbreviations: Ca, calcium; IP3, inositol triphosphate; IP4, inositol tetraphosphate; IP5, inositol pentaphosphate; IP6, inositol hexaphosphate; NE, necrotic enteritis; phy, phytase.

### Fluorescein Isothiocyanate Dextran, Day 16

Challenge birds had higher (*P* < 0.05) FITC-d detected in the blood than the unchallenged birds (Table 6). No interactions or effects of dietary treatment were observed.

**Table 6:** Effect of necrotic enteritis, phytase, and calcium on gut permeability, day 16 posthatch.

Effects				FITC-d (μg/mL)
	NE	Phy	Ca	
Main effects				
NE	-			0.03^b^
	+			0.34^a^
SEM				0.12
P > f				
NE				0.000
Phy				0.821
Ca				0.635
NE × Phy				0.939
NE × Ca				0.520
Phy × Ca				0.637
NE × Phy × Ca				0.418

^a,b,c^Means in the same column with different superscripts are significantly different (*P* < 0.05).

Phytase (Quantum Blue 5G).

2-way or 3-way interaction by Tukey.

Abbreviations: Ca, calcium; FITC-d, fluorescein isothiocyanate dextran; NE, necrotic enteritis; phy, phytase.

### Gene Expression, Day 16

Only genes that showed a tendency or significant response have been reported in this article. Those which showed a muted response: tight junction protein 1, calcium channel, voltage-dependent, beta 1 subunit, calcium channel, voltage-dependent, gamma subunit 1, solute carrier family 8 (sodium/calcium exchanger), member 1, and two-pore segment channel 1 are presented as Supplementary Data.

A phytase × Ca interaction was detected for vitamin D receptor (**VDR**) expression, where low level of Ca downregulated the expression of VDR only in birds receiving the diet supplemented with low phytase (Table 7). A phytase by Ca interaction was observed for CLDN1 expression; birds on the low phytase and low Ca diet recorded higher expression (*P* < 0.001) compared with those on any other treatment, and birds fed low Ca with the high phytase dose or high Ca with the low phytase dose had lower CLDN1 expression than those on any other treatment. In the group fed high phytase, high Ca tended (*P* = 0.052) to increase the expression of *MUC2.* The challenge as main effect downregulated the expression of MUC-2 (*P* < 0.05) NaPi-IIb (*P* < 0.001), occludin (**OCLD**) (*P* < 0.001), and VDR (*P* < 0.01) (Table 7). The challenge however upregulated the expression of CLDN1 (*P* < 0.001) and CLDN5 (*P* < 0.001). A 3-way challenge, phytase and Ca interaction was detected for calcium-sensing receptor (**CASR**) where the unchallenged birds fed low phytase and low Ca exhibited a higher expression of CASR than all other groups (Table 7). The challenge as main effect downregulated the expression of ATPase Na+/K+ transporting subunit alpha 1 (*P* < 0.001), ATP15 (*P* < 0.001), Calbindin 1 (**CALB1**) (*P* < 0.001), ATPase, Ca++ transporting, cardiac muscle, fast twitch 1 (*P* < 0.004), ATPase, Ca++ transporting, plasma membrane 1 (*P* < 0.001), (sodium/lithium/calcium exchanger), member B1 (**SLC8B**) (*P* < 0.003), 2-pore segment channel 2 (**TPCN2**) (*P* < 0.001), and TPCN (*P* < 0.001) (Table 8). The challenge strongly tended to upregulate ATPase type 13A4 (*P* < 0.051). High phytase tended to downregulate the expression of calcium channel, voltage-dependent (**CACNA**).

**Table 7:** Effect of necrotic enteritis, phytase, and calcium on jejunal gene expression, day 16 posthatch.

Effects				CLDN1	CLDN5	JAM2D	MUC-2	NaPi-IIb	OCLD	VDR
	NE	Phy	Ca							
2-Way interactions										
Phy∗Ca										
		500	Low	1.379^a^	1.191	1.006	1.061	1.669	1.099	1.740^a^
		500	High	0.773^c^	1.234	1.120	1.002	1.188	1.029	0.940^b^
		1,500	Low	0.864^c^	0.997	0.999	0.965	1.285	1.115	0.930^b^
		1,500	High	1.014^b^	1.192	1.043	1.365	1.187	1.011	1.050^b^
Main effects										
NE	-			0.513^b^	0.771^b^	0.974	1.247^a^	1.787^a^	1.294^a^	1.500^a^
	+			1.502^a^	1.537^a^	1.110	0.949^b^	0.878^b^	0.832^b^	0.830^b^
Ca			Low	1.121	1.094	1.002	1.013	1.477	1.106	1.330
			High	0.893	1.213	1.081	1.184	1.187	1.020	1.000
SEM				0.45	0.46	0.21	0.26	0.36	0.21	0.55
P > f										
NE				0.000	0.000	0.115	0.013	0.000	0.000	0.003
Phy				0.435	0.506	0.621	0.251	0.279	0.999	0.112
Ca				0.199	0.502	0.358	0.146	0.106	0.287	0.120
NE × Phy				0.872	0.394	0.072	0.341	0.760	0.451	0.599
NE × Ca				0.552	0.658	0.060	0.382	0.467	0.613	0.203
Phy × Ca				0.036	0.667	0.683	0.052	0.280	0.820	0.038
NE × Phy × Ca				0.137	0.756	0.142	0.504	0.533	0.988	0.934

^a,b,c^Means in the same column with different superscripts are significantly different (*P* < 0.05).

Phytase (Quantum Blue 5G).

2 or 3-way interaction by Tukey.

Abbreviations: Ca, calcium; CLDN1, claudin 1; CLDN5, claudin 5; JAM2D, junctional adhesion 2; MUC-2, mucin 2; NE, necrotic enteritis; NaPi-IIb, Na-dependent Pi cotransporters, type IIb; phy, phytase; OCLD, occluding; VDR, vitamin D.

**Table 8:** Effect of necrotic enteritis, phytase, and calcium on jejunal gene expression (Cont.), 16 posthatch.

Effects				CALB1	ATP1A1	ATP5A1W	ATP13A4	ATP2A1	ATP2B1	CACNA	CASR	SLC8B	TPCN2	TPCN3
	NE	Phy	Ca											
Treatment means														
1.	-	500	Low	2.888	1.400	1.276	0.878	1.117	1.603	1.345	2.133^a^	1.921	1.463	1.283
2.	-	1,500	Low	1.835	1.458	1.271	0.908	1.047	1.247	0.900	1.158^b^	1.549	1.266	1.398
3.	-	500	High	2.081	1.269	1.121	0.946	1.112	1.271	1.047	0.796^b^	1.488	1.283	1.238
4.	-	1,500	High	1.365	1.565	1.344	0.979	1.237	1.334	1.020	0.917^b^	2.177	1.403	1.463
5.	+	500	Low	0.914	0.716	0.848	1.455	0.872	0.960	1.440	1.009^b^	0.815	0.966	0.807
6.	+	1,500	Low	1.072	0.817	0.812	0.928	0.880	0.854	0.983	1.026^b^	0.584	0.839	0.810
7.	+	500	High	0.863	0.759	0.864	1.207	0.912	0.783	0.988	1.074^b^	1.124	0.869	0.792
8.	+	1,500	High	0.624	0.760	0.864	1.268	1.062	0.862	0.909	0.860^b^	1.037	0.798	0.746
Main effects														
NE	-			1.423^a^	1.253^a^	2.042^a^	0.928^b^	1.128^a^	1.364^a^	1.078	1.251	1.784^a^	1.354^a^	1.345^a^
	+			0.763^b^	0.847^b^	0.868^b^	1.214^a^	0.932^b^	0.865^b^	1.080	0.992	0.890^b^	0.868^b^	0.789^b^
Phy		500		1.036^b^	1.027	1.686	1.121	1.003	1.154	1.205^a^	1.253	1.337	1.145	1.030
		1,500		1.150^a^	1.073	1.224	1.021	1.056	1.074	0.953^b^	0.990	1.337	1.077	1.104
Ca			Low	1.677^b^	1.098	1.052	1.042	0.979	1.166	1.167	1.332	1.217	1.133	1.074
			High	1.233^a^	1.088	1.048	1.100	1.081	1.063	0.991	0.912	1.456	1.088	1.060
SEM				0.59	0.26	0.17	0.32	0.12	0.28	0.28	0.31	0.54	0.28	0.24
P > f														
NE				0.000	0.000	0.000	0.051	0.004	0.000	0.989	0.040	0.003	0.000	0.000
Phy				0.075	0.216	0.538	0.485	0.415	0.531	0.064	0.037	1.000	0.590	0.408
Ca				0.087	0.917	0.962	0.687	0.125	0.421	0.191	0.001	0.400	0.722	0.868
NE × Phy				0.104	0.492	0.393	0.360	0.690	0.604	0.903	0.185	0.575	0.812	0.286
NE × Ca				0.448	0.976	0.612	0.933	0.889	0.880	0.516	0.004	0.616	0.851	0.780
Phy × Ca				0.953	0.707	0.374	0.306	0.201	0.240	0.140	0.083	0.290	0.464	0.865
NE × Phy × Ca				0.473	0.359	0.516	0.311	0.838	0.647	0.940	0.010	0.418	0.608	0.658

^a,b,c^Means in the same column with different superscripts are significantly different (*P* < 0.05).

Phytase (Quantum Blue 5G).

2-way or 3-way interaction by Tukey.

Abbreviations: ATP1A1, ATPase Na+/K+ transporting subunit alpha 1; ATP5A1W, ATP synthase subunit alpha; ATP13A4, ATPase type 13A4; ATP2A1, ATPase, Ca++ transporting, cardiac muscle, fast twitch 1; ATP2B1, ATPase, Ca++ transporting, plasma membrane 1; Ca, calcium; CALB1, Calbindin 1; CACNA, calcium channel, voltage-dependent, P/Q type alpha 1A subunit; CASR, calcium-sensing receptor; NE, necrotic enteritis; phy, phytase; SLC8B, (sodium/lithium/calcium exchanger), member B1; TPCN2, 2-pore segment channel 2; TPCN3, 2-pore calcium channel 3.

### Morphometric Measurements of Jejunum

A Ca × challenge interaction was detected for basal width of jejunal villi (*P* < 0.05), where basal width was increased in birds challenged and fed low Ca compared with the unchallenged birds or challenged birds fed high Ca (Table 9). The challenge as a main effect decreased the total height (muscularis layer + villi height) of the mucosa (*P* < 0.001), villi height (*P* < 0.001), crypt and villi ratio (*P* < 0.001), and apparent villi surface area (*P* < 0.001) (Table 9). The challenge also increased crypt depth (*P* < 0.01) and apical width of villi (*P* < 0.001) and basal width of villi (*P* < 0.01).

**Table 9:** Effect of necrotic enteritis, phytase, and calcium on intestinal morphology, 16 posthatch.

Effects				Total height^1^ (μm)	Muscularis layer (μm)	Crypt depth (μm)	Villi height (μm)	Basal width (μm)	Apical width (μm)	Crypt:villi	Apparent villi area (μm^2^ × 10^3^)
	NE	Phy	Ca								
2-Way interactions											
NE∗Ca											
	-		Low	1,468.8	206.5	184.1	1,078.2	57.44^b^	37.15	6.28	38.50
	-		High	1,472.2	183.2	189.5	1,099.6	57.20^b^	34.55	6.17	38.13
	+		Low	886.6	164.8	256.8	465.0	69.78^a^	54.22	2.10	41.20
	+		High	1,053.8	210.8	283.6	559.4	58.83^b^	45.98	2.38	40.39
Msin effects											
NE	-			1,470.5^a^	194.8	186.8^a^	1,088.9^a^	57.32	35.85^b^	6.22^a^	50.67^a^
	+			970.2^b^	187.8	270.2^b^	512.2^b^	64.31	50.10^a^	2.24^b^	28.43^b^
Ca			Low	1,177.7	185.6	220.4	771.6	63.61	45.68	4.19	39.85
			High	1,263.0	197.0	236.5	829.5	58.01	40.27	4.28	39.26
SEM				187.76	37.59	65.31	166.12	5.61	7.51	1.25	7.48
P > f											
NE				0.000	0.713	0.005	0.000	0.006	0.000	0.000	0.000
Phy				0.801	0.442	0.823	0.476	0.475	0.672	0.649	0.413
Ca				0.256	0.552	0.569	0.301	0.027	0.055	0.851	0.846
NE × Phy				0.976	0.798	0.400	0.634	0.481	0.785	0.636	0.608
NE × Ca				0.275	0.074	0.704	0.512	0.033	0.310	0.684	0.926
Phy × Ca				0.876	0.897	0.885	0.928	0.902	0.305	0.658	0.942
NE × Phy × Ca				0.532	0.197	0.653	0.136	0.167	0.222	0.262	0.071

^a,b,c^Means in the same column with different superscripts are significantly different (*P* < 0.05).

2-way or 3-way interaction by Tukey.

Phytase (Quantum Blue 5G).

1 Total height (muscularis layer + villi height).

## Discussion

This study investigated the interactive effect of dietary Ca and phytase on gut permeability, phytate esters degradation, jejunal gene expression, and intestinal morphology of broilers during subclinical NE. The application of exogenous phytases aims to hydrolyze phytate (IP6) and its intermediate derivatives. The effects of IP6 include the increase in endogenous losses of proteins and a decrease in the rate of conversion of pepsinogen to pepsin (Yu et al., 2012). In the present work, high phytase increased IP3 in the gizzard, whereas it decreased IP4 and IP5. These findings, therefore, support the consensus that higher doses of phytase are beneficial in promoting performance because they reduce the antinutritive effect of phytate with the generation of more soluble lower phytate esters and inositol (Cowieson et al., 2013; Gautier et., 2018). The accumulation of IP4 and IP3 with the use of high phytase has been previously shown, and the conditions of the current study (Ca and P levels, age, breed, etc) might influence the degree of accumulation of each ester. Higher doses of phytase have been shown to remove this accumulation, and in the current study, it is clear that the combination of the high dose of phytase with a low concentration of calcium reduces the accumulation to the point that it is numerically lower than the 500 dose in the ileum. This observation was also noted by Walk et al. (2018) who detected a reduction in IP6 and IP5 in gizzard digesta when phytase increased from 500 to 1,500 FTU/kg. In fact, the concentration of IP6 in the 500 FTU treatment in the current study and that of Walk et al. (2018) were similar, and the reduction between 500 and 1,500 somewhat similar as well, perhaps the more limited reduction noted in the present study relates to our main effect of high dose being summed over the 2 levels of calcium, the higher level tending to elevate IP6 levels 2 fold. Walk et al. (2018) also observed an increase in IP3, but only when phytase increased from 500 and 1,500 FTU/kg and then a decline at a concentration of 4,500 FTU/kg. This confirmed the report of Tamim et al. (2004) showing that the majority of IP degradation happens in the upper GIT segments, where the impact of IP6 and Ca chelation is less negative to phytase efficacy. Walk et al. (2018) concluded that supplementation of broiler diets with phytase up to 4,500 FTU/kg was beneficial to weight gain and resulted in nearly complete phytate and phytate ester destruction.

The findings of the current study corroborate with many reports that even in diets supplemented with phytase, the incremental concentration of Ca increases residual concentration not only of IP6 but also IP5, IP4, and IP3 as well (Zeller et al., 2015; Li et al., 2016; Beeson et al., 2017). Bedford and Rousseau (2017) had argued that a severely imbalanced diet (in the case of this work, a wide Ca:P) might compromise the response of high dietary phytase because such imbalances inhibit the conversion of IP6 to the lower esters. The results therefore suggest that to dephosphorylate IP6 ultimately to inositol, high levels of phytase with a low dietary Ca level (or narrower Ca:P) is recommended. The mechanism by which high dietary Ca and wider Ca:P impairs phytate degradation is that excessive Ca precipitates phytate into insoluble Ca-phytate complexes. This Ca-phytate formation is influenced by the pH coupled and Ca:phytate molar ratios. Thus, the higher pH recorded in the ileum relative to higher dietary Ca at day 29 in the present study (Zanu et al., 2020a) might have contributed to the reduction in the degradation of phytate. Several experiments generally support the observation that higher pH is pivotal to Ca-phytate complex formation (Tamim et al., 2004; Li et al., 2017). The current study indicates that high phytase with low Ca is recommended, as the combination was able to decrease the higher phytate esters. In addition, a higher level of Ca appeared to increase the concentration of IP5 and IP6 in the ileum (regardless of NE challenge), suggesting that the higher ileal pH observed in this study at day 29 (Zanu et al., 2020a) as a result of high dietary Ca might have caused precipitation of these esters. The reason is that the phytase used produces IP1 and not inositol may be erroneously stated in other reports. Alkaline phosphatase in the small intestinal mucosa is likely responsible for removal of the last P from IP1 to produce inositol. Hence, it is possible that increased inositol observed in the gizzard in other phytase dose studies is a result of reflux from the duodenum.

The intestinal mucosa plays a key role in nutrient digestion, absorption, and defense against pathogen infection in the host (Sanchez de Medina et al., 2014). Several reports support the fact that enteric inflammation compromises the tight junctions function and increase permeability (Vicuña et al., 2015a, 2015b; Kuttappan et al., 2015; Barekatain et al., 2019). An indication of damaged intestinal mucosa and disruption of the intestinal tight junctions in the present study is the higher concentration of the FITC-d marker in the serum observed in the challenge group.

The intestinal inflammatory response to intestinal coccidia leads to the secretion of mucus which serves as a substrate for *C. perfringens* (Collier et al., 2003). The most abundant mucin-producing gene in the intestine is the gel-forming mucin (***MUC2***) that was measured in this study. The findings on the expression of jejunal *MUC2* in this study agrees with other studies; that is, challenge downregulates the expression of *MUC2* (Forder et al., 2012; Oh et al., 2018). Those authors reported lower MUC2 *mRNA* levels in NE-unchallenged birds treated with antibiotics. The downregulation of *MUC2* in the present study because of challenge (with no antibiotic treatment) was rather unexpected as more mucin was needed to defend the mucosa. A recent study did also report the downregulation of *MUC2* in the jejunum due to NE (Zanu et al., 2020b). It could be conjectured that as the intestine deteriorates, *MUC2* expression decreases, slowing the replenishment of the mucus layer. The high expression of *MUC2* in birds fed high phytase and high Ca diet might have reduced phytase activity resulting in the formation of Ca-phytate complexes, thereby increasing gastric secretions. An increase in gastric secretions likely led to an increase in mucin production in the intestine. The increase in mucin because of excessive gastric secretion is necessary to protect the GI tract from digestion by the enzymes and HCl (Rutherfurd et al., 2004; Walk and Olukosi, 2019). Another possibility for the higher expression of *MUC2* is an interaction of Ca-phytate complex with the protein digestion process that would trigger MUC2 expression. The expression would possibly occur in an area where the condition is not within the phytase effective activity range and preferentially in the presence of high Ca; thus, Ca had prior access to enable the formation of complex probably before phytase could interact with the phytate, impeding the phytase effectiveness.

Claudins and occludins are the 2 of the 3 integral membrane proteins which define the barrier structure of the paracellular pathway (Gil-Cardoso et al., 2016). Therefore, the regulation of the expression of these proteins is crucial in reducing gut disorder. In this study, the NE challenge led to the upregulation of the expression of the claudins (CLDN1 and CLDN5) but downregulated the expression of OCLD. Claudins determine the permeability of the space between 2 adjacent cells (Fuladi et al., 2020). Therefore, a higher expression of claudins in the current study is an indication of a response of the birds to try to repair the damage that was evident because of the increased FITC-d. The downregulation of OCLD as a result of the challenge agrees with those of other studies (Wang et al., 2017; Li et al., 2018), but it contradicted what was reported by Knight et al. (2016). The difference in the expression of claudins and OCLD is not unusual as according to Lee (2015) claudins do not have any sequence similarity to OCLD.

Furthermore, some studies suggest that damage to the intestinal epithelium decreases the effectiveness of nutrient transporter as well as their expression (Fetterer et al., 2014; Guo et al., 2014). Calcium transporting genes were of major interest in this study. All the nutrient transporter genes (CALB1, ATP1A1, ATP5A1W, ATPase, Ca++ transporting, cardiac muscle, fast twitch 1, ATPase, Ca++ transporting, plasma membrane 1, SLC8B, TPCN2 and TPCN3, NaPi-IIb, and VDR) measured in this study were downregulated by the NE challenge. Transcellular Ca absorption takes place in 3 stages: first, transport through the epithelial voltage-insensitive channels Ca channels into the enterocyte; second, intracellular transcytosolic diffusion by calcium-binding proteins (calbindin), and third, extrusion out of the cell to blood vessels by ATP-activated basolateral membrane Ca pump and by the Na^+^/Ca^2+^ exchanger NCX1 (Kiela and Ghishan, 2016). The Ca channel, CACNA, was the only gene that responded to phytase, that is, it was downregulated by high phytase. The expression of transporters of Ca is high during states in which a high efficiency of calcium absorption is required and vice versa. It implies that high phytase might have released more Ca in the intestine and hence the lower expression because of lesser need. Though solute carrier family 8 member 1 (SLC8B) and two-pore segment channels (TPCN2 and TPCN3) have been reported to aid calcium absorption in the intestine (Lee and Kim, 2018), they did not respond to dietary Ca level in the current study. Rather, they were downregulated by the challenge. Many nutrient transporters are located on the tip of the villi, and so villi atrophy and sloughing of the brush border membrane of the enterocytes as was detected in the current study is likely to reduce the transporters agreeing with the reports of other studies (Guo et al., 2013; Leung et al., 2019). Perhaps in addition to the reduced brush border, the enterocytes themselves were less mature and thus expressed fewer transporters.

Following Ca uptake through the above channels, calbindin D_28k_ protein binds to Ca and facilitates its movement to the basolateral membrane. In the present study, high dietary Ca tended to reduce the expression of calbindin D_28k_ protein (CALB1). Reduction in dietary Ca was also reported to have led to the upregulation of the expression of calbindin (Li et al., 2012). In that study (Li et al., 2012), the calcium sources were limestone and dicalcium phosphate, and the 2 dietary calcium levels were 1.10 and 0.60%, and 2 dietary nPP levels were 0.50 and 0.27%. The last stage of Ca absorption is the pumping of the Ca2+ ions out of the cytosol into the external medium with the help of ATPases. Contrary to the hypothesis of this study, all the ATPases measured in this study, except ATPase type 13A4 were downregulated because of the challenge while none of them responded to dietary Ca or phytase levels. The reason for the downregulation of ATPases in the challenged birds in the present study might be that inflammation of the jejunum could have led to fluid and electrolyte loss from the cytosol, and hence, ATPases activities and pathways were preserved. Conversely, these observations could have been simply because of sloughing of the brush border, thus reducing the number of the cells that secrets these enzymes.

Further, CASR play a key role in regulating calcium homeostasis in chickens. The Ca^2+^ is a multipurpose messenger that exerts its effects inside and outside the cell. Through CASR, the parathyroid gland maintains serum Ca^2+^ concentrations within a very narrow physiological range by modulating the minute-to-minute release of parathyroid hormone into the circulation. In the present work, unchallenged birds fed low phytase and low Ca exhibited a higher expression of CASR. It appears that a low phytase in the presence of a lower Ca led to a lower Ca concentration in the lumen of the intestine and hence a corresponding lower expression of CASR. The possible low Ca concentration in the intestine agrees with the result on serum Ca where lower dietary Ca led to a lower serum Ca^2+^ in unchallenged birds (Zanu et al., 2020c). Furthermore, in the present study, low phytase led to the upregulation of CACNA. It appears that phytase supplementation at a lower concentration upregulates the expression of calcium-related genes in broilers (Zanu et al., 2020b). It could be speculated that a lower phytase supplementation means lower calcium released from the diets; hence, the birds' response to increasing the expression of the related transporting genes.

In addition to CASR, the physiological role vitamin D_3_ plays in Ca homeostasis in chickens has been studied extensively. In the current study, birds on low Ca and low phytase resulted in increased expression of VDR and might have probably resulted in the lowest Ca content in the intestine and blood hence the incremental VDR expression which is likely a response to try to optimize Ca absorption. The downregulation of VDR in the challenged birds in the present study is also noteworthy. The downregulation of VDR in the challenged birds could be likely because of a lower Ca intake during the challenge. For instance, reduced Ca intake has been associated with diminished VDR mRNA in avians parathyroid gland (Russell et al., 1993). A lower expression of VDR is also reported in many gut-related diseases in chickens and in other species (Wu et al., 2015; Garg et al., 2019).

In addition to the Ca transporting genes, the challenge downregulated the expression of NaPi-IIb which is involved in regulating both intestinal Pi (phosphate) absorption and renal Pi resorption. The type IIb cotransporter is primarily expressed in the brush-border membranes of the small intestinal epithelium, where it is the major Na-Pi cotransporter. An upregulation of NaPi-IIb was also reported in coccidial vaccine challenge birds (Adedokun and Adeola, 2016).

The effect of NE on decreasing villi height and increasing crypt depth has also been reported in other studies (Van Immerseel et al., 2018; Xue et al., 2018). Further, the challenge group recorded wider basal and apical widths similar to the observation of Chen et al. (2015). Those authors explained that narrower villi have greater nutrient absorption area and that widening of the villus indicates less nutrient absorption area and probably also a greater amount of gut-associated immune tissue proliferation and accumulation in the villus, which is another indication of compromised gut health. A reduction in nutrient uptake and growth performance is the result of poor gut morphology which is a common feature in challenged birds. Longer villi as seen in the unchallenged birds in this study is reported to be correlated with increased villus surface area (Bakare and Chimonyo, 2017). Therefore, a decrease in villus surface area in the challenged birds of the trial means a decrease in the absorptive area and less capacity for absorption of nutrients with consequent effect on performance.

In conclusion, while phytase, especially at a higher inclusion rate, increased the hydrolysis of phytate esters, excessive dietary Ca might counter this benefit. It appears that greater higher-ester degradation is associated with improved performance and digestibility and given degradation was not complete a higher dose *may* have been beneficial. In addition, the present data suggest that high Ca and high phytase, while not the best for IP6 destruction, did not lead to huge reductions in indicators of gut health. The challenge generally impaired the intestinal morphological traits with consequent depression of most nutrients transporting inflammatory and tight junction protein genes, thus stressing the call for a search for measures to reduce NE incidence in broiler flock.
