# Supplementary material for: Interactive effect of dietary calcium and phytase on broilers challenged with subclinical necrotic enteritis: part 2. Gut permeability, phytate ester concentrations, jejunal gene expression, and intestinal morphology
Source: Poult Sci. 2020 Jul 3;99(10):4914–28. doi: 10.1016/j.psj.2020.06.030 (PMC7598120; doi:10.1016/j.psj.2020.06.030)
Supplement: Supplementary Data [file mmc1.docx]

**Supplementary data**

Table 10. Effect of necrotic enteritis, phytase and calcium on jejunal gene expression, 16 post-hatch

| Effects | | | | TJP1 | CACNB | CACNG | SLC8A | TPCN1 |
| --- | --- | --- | --- | --- | --- | --- | --- | --- |
|  | NE | Phy | Ca |  |  |  |  |  |
| Main effects |  |  |  |  |  |  |  |  |
| NE | - |  |  | 1.108 | 1.082 | 1.140 | 1.025 | 0.998 |
|  | + |  |  | 1.056 | 1.029 | 1.140 | 1.164 | 1.056 |
|  |  |  |  |  |  |  |  |  |
| Phy |  | 500 |  | 1.083 | 1.024 | 1.074 | 1.110 | 1.033 |
|  |  | 1500 |  | 1.080 | 1.088 | 1.206 | 1.079 | 1.022 |
|  |  |  |  |  |  |  |  |  |
| Ca |  |  | Low | 1.091 | 1.032 | 1.088 | 0.987 | 1.018 |
|  |  |  | High | 1.073 | 1.079 | 1.192 | 1.202 | 1.036 |
| SEM |  |  |  | 0.25 | 0.21 | 0.33 | 0.25 | 0.12 |
| P>f |  |  |  |  |  |  |  |  |
| NE |  |  |  | 0.693 | 0.599 | 1.000 | 0.292 | 0.426 |
| Phy |  |  |  | 0.987 | 0.530 | 0.456 | 0.817 | 0.880 |
| Ca |  |  |  | 0.884 | 0.641 | 0.556 | 0.104 | 0.802 |
| NE × Phy |  |  |  | 0.519 | 0.972 | 0.335 | 0.784 | 0.354 |
| NE × Ca |  |  |  | 0.849 | 0.372 | 0.909 | 0.120 | 0.491 |
| Phy × Ca |  |  |  | 0.242 | 0.161 | 0.790 | 0.459 | 0.276 |
| NE × Phy × Ca |  |  |  | 0.795 | 0.655 | 0.905 | 0.995 | 0.867 |

^a,b,c^means in the same column with different superscripts are significantly different (P < 0.05)

Abreviations: NE, necrotic enteritis; phy, phytase; Ca, calcium; TJP1, Tight junction protein 1; CACNB, calcium channel, voltage-dependent, beta 1 subunit; CACNG, calcium channel, voltage-dependent, gamma subunit 1SLC8A, solute carrier family 8 (sodium/calcium exchanger), member 1 and TPCN1, two-pore calcium channel 3

Phytase (Quantum Blue 5G)

2 or 3-way interaction by Tukey
